# Addressing the challenges of applying precision oncology

**DOI:** 10.1038/s41698-017-0032-z

**Published:** 2017-09-04

**Authors:** Seung Ho Shin, Ann M. Bode, Zigang Dong

**Affiliations:** 1 The Hormel Institute, University of Minnesota, Austin, MN 55912 USA; 2 Program in Bioinformatics and Computational Biology, University of Minnesota, Minneapolis, MN 55455 USA

## Abstract

Precision oncology is described as the matching of the most accurate and effective treatments with the individual cancer patient. Identification of important gene mutations, such as *BRCA1/2* that drive carcinogenesis, helped pave the way for precision diagnosis in cancer. Oncoproteins and their signaling pathways have been extensively studied, leading to the development of target-based precision therapies against several types of cancers. Although many challenges exist that could hinder the success of precision oncology, cutting-edge tools for precision diagnosis and precision therapy will assist in overcoming many of these difficulties. Based on the continued rapid progression of genomic analysis, drug development, and clinical trial design, precision oncology will ultimately become the standard of care in cancer therapeutics.

## Introduction

Improving efficacy, minimizing the adverse side effects of drugs, and overcoming acquired resistance to drug treatment have been major goals and emphases in cancer therapy. In order to attain the objectives of precision oncology, basic and clinical researchers have identified and clarified differences derived from genetic features between individuals. The existence of specific genetic differences between individuals is exemplified by the finding in 1932 that phenylthiocarbamide (PTC) exhibits the unusual property of either tasting very bitter or having no taste at all based on the individual’s genetics. Notably, this trait is inherited to the next generation.^1, 2^ The “one-size-fits-all strategy” is no longer relevant to cancer treatment. The tailoring of distinct treatments to each specific individual became known as “personalized medicine”^3^ and later the name was changed to “precision medicine”.^4^ Many countries have now launched government-driven projects focusing on precision medicine, including the Precision Medicine Initiative (National Institutes of Health, USA),^4^ Cancer Moonshot program^5^ (National Cancer Institute, USA) and HORIZON 2020 Work Program for 2016–2017 (EU).^6^

The accelerating momentum of precision medicine, and especially precision oncology, has stemmed from the increasing amount of “-omics” information acquired from patients and, importantly, the successful integration of the fields of basic and clinical cancer research. Next-generation sequencing instrumentation is capable of sequencing several genomes a day at a cost of about $1000 each, making this technology an essential and straightforward part of translational cancer research.^7^ Driver-gene mutations identified from comprehensive genome analyses are now frequently detected in many cancer patients,^8^ and the aberrant gene products are currently being targeted by specific antagonists or monoclonal antibodies.^9^ Oncologists are now able to stratify subsets of cancer and make informed therapeutic decisions. Consequently, targeted therapy has gained credibility in reinforcing and/or replacing conventional cytotoxic chemotherapy. Several targeted agents are presently approved by the FDA and are being used clinically against several types of cancer.

Here, we categorize the work flow of precision oncology into two segments, precision diagnosis and precision therapy, and provide milestones and important aspects characterizing each segment. By reviewing targeted therapies clinically approved against breast cancer, lung cancer and melanoma, we reveal the current status and expose possible challenges in precision oncology. Two of the most effective state-of-the-art tools for the success of precision oncology are also described.

## Precision oncology for patients—from precision diagnosis to precision therapy

In order to better understand and utilize precision oncology (i.e., precision medicine as it applies to cancer), analyzing the procedures step by step is crucial. Precision oncology comprises precision diagnosis and precision therapy. Precision diagnosis begins with an accurate diagnosis of each individual cancer patient and ideally classifies subjects into cancer patients and individuals at high risk for specific cancers.^10^ By detecting biomarkers that are associated with specific cancer types such as *BRCA1/2* mutations in breast cancer,^11, 12^ we can diagnose the current or potential risks of each individual. Accumulating evidence shows that multiple biomarkers (so called “signature”) can help in creating more precise and evidence-based therapeutic strategies to modulate cancer.^13, 14^ Establishing molecular subtypes and categorizing tumors into one of the subtypes enhances the accuracy of therapeutic options.^15^ The information derived from precision diagnosis reveals the precise medical measures, including surgery, radiation, chemotherapy, adjuvant therapy, supplements, and/or vaccines,^16^ that are needed for each individual.

Precision therapy matches the most effective treatment to the individual cancer patient based on the genetic profile of the specific cancer, and can be divided into two categories that include precision chemotherapy and precise therapeutic procedures. Precision chemotherapy is the use of the correct drugs prescribed that assures maximum benefit with minimum risk or toxicity for the patient. Therapeutic measures could include surgery and radiation therapy tailored to the patient’s needs.

### Precision diagnosis

Information-based diagnosis can assist clinicians not only in identifying tumor type and stage, but also in revealing important genetic mutations that drive carcinogenesis. Advances in technology have clearly resulted in more effective therapeutic decisions. Final goals include optimization of clinical outcomes, avoidance of unnecessary therapies, minimized side effects, and overcoming or avoiding drug resistance.

#### BRCA1/2 are a milestone for precision diagnosis

The discovery of the breast cancer susceptibility genes, *BRCA1/2*, was traced from a family with a history of breast cancer, and indicated the association between genetic features and the early onset of the disease.^11, 12^ In addition to other breast cancer susceptibility genes, the *BRCA1/2* mutation test is one of the most well-established models in precision oncology. It has become a guideline that aids clinicians in creating prevention strategies and targeted therapies. More than one million individuals have been tested for *BRCA1/2* mutations worldwide.^17^

The landscape of mutations in the *BRCA1/2* genes has been extensively studied and the relationship between the mutations and breast cancer risk is also well-defined. More than 1800 different variants (i.e., intronic changes, insertions, deletions and missense mutations) have been observed in *BRCA1* and 2000 different variants have been reported to occur in *BRCA2*.^17^ *BRCA1*/2 mutations are currently the most significant gene variations in breast cancer surpassing tumor protein p53 (*TP53*), phosphatase and tensin homolog (*PTEN*), liver kinase B1 (*LKB1*), and cadherin1 (*CDH1*) mutations.^17–19^ *BRCA1/2* mutations are estimated to account for ~15% of the relative familial risk of breast cancer.^17^ Mutations in *BRCA1* and *BRCA2* reportedly contribute equally to early-onset breast cancer.^20^

#### Development and improvement of genetic tests

Recent progress in immunohistochemistry (IHC) has helped clinicians to identify the presence of specific biomarkers and to categorize patients in pathology.^21, 22^ Detection of human epidermal growth factor receptor 2 (HER2)^23^ in breast cancer and PD-L1^24^ in lung cancer based on IHC aided the prescription of suitable drugs for the patients. However, many other techniques are currently available to quantify changes in gene expression, and include reverse transcriptase-polymerase chain reaction,^25^ DNA arrays,^26^ NanoString technology,^27^ comparative genomic hybridization arrays, and single-nucleotide polymorphism analysis.^28^ Genetic tests have been developed for diagnostic, predictive and prognostic purposes and some have been approved by the FDA, whereas others are still under development for breast cancer,^29–37^ lung cancer,^38–44^ and melanoma^45–49^ (Table 1). Although the most significant genes, such as *BRCA1*/2, can only predict or explain a portion of disease susceptibility, the number of genes examined in a single test has continually increased to improve accuracy.^14, 50^ Of particular note (Table 1), the 70-gene signature test for breast cancer (MammaPrint by Agendia)^29^ showed the most efficacy in a clinical trial.^51^ The study in 6693 women with early-stage breast cancer was conducted to examine whether the gene signature test could reduce the use of chemotherapy. For patients who had high clinical risk and low genomic risk for recurrence, the difference in the 5-year survival rates between chemotherapy (98.8%) and no-chemotherapy (97.3%) was only 1.5%.^51^ The result shows that approximately 46% of women with breast cancer who are at high clinical risk might not require chemotherapy and that the 70-gene signature could aid in treatment decisions.^51^ The Oncotype Dx test by Genomic Health also helps clinicians in selecting proper treatment options for patients with invasive breast cancer.^34, 52^ The Oncotype Dx test generates a recurrence score (0–100) by analyzing the expression of 21 genes.^53, 54^ Survival rates of patients with high recurrence scores (31 and higher) have been improved by adjuvant chemotherapy, whereas patients with low recurrence scores (less than 17) are unlikely to get benefits from the chemotherapy.^52^

**Table 1:** Genetic tests for breast cancer, lung cancer and melanoma

Type	Test name	Institution	FDA approval	No. of genes	Ref.
Breast cancer	MammaPrint	Agendia	Yes	70	29
	Prosigna	NanoString Tech.		50	33
	GeneSearch BLN test	Veridex		2	30
	INFORM HER2 Dual ISH	Ventana		1	32
	HER2 CISH pharmDx Kit	Dako		1	31
	Oncotype Dx	Genomic Health	No	21	34
	EndoPredict	Sividon Diagnostics		11	35
	Breast Cancer Index test	BioTheranostics		7	36
	FEMTELLE	Sekisui Diagnostics		2	37
Lung cancer	Therascreen EGFR RGQ PCR kit	QIAGEN	Yes	1	40
	cobas EGFR Mutation Test	Roche		1	38
	VENTANA ALK (D5F3) CDx Assay	Ventana Medical Systems		1	41
	Vysis ALK Break Apart FISH Probe Kit	Abbott Molecular		1	39
	Lung Cancer Mutation Panel	Quest Diagnostics	No	34	42
	Lung Cancer Comprehensive Mutation and Translocation Panel	ARUP Laboratories		11	43
	SnaPshot Multiplex System	Thermo Fisher		11	44
Melanoma	cobas 4800 BRAF V600	Roche	Yes	1	45
	THxID-BRAF	bioMerieux		1	46
	53-Immune Gene Network Panel	Icahn School of Medicine Mount Sinai	No	53	47
	myPATH	Myriad		23	48
	Sentosa SQ Melanoma Panel	Vela Diagnostics		10	49

### Precision therapy

Precision therapies have been applied in breast cancer, lung cancer and melanoma, but many challenges still need to be addressed.

#### Breast cancer and targeted therapy

The most well-known target-based treatment against breast cancer is directed at the estrogen receptor (ER) and the HER2 (Table 2).^55^ The discovery of these two protein receptors opened a new avenue for targeted therapy that showed improved efficacy compared to aromatase inhibitors, which suppress plasma estrogen levels in postmenopausal women.^56^ Tamoxifen, a pro-drug targeting the ER, is metabolized in the liver into active metabolites that have a higher affinity for the ER compared to the parental tamoxifen.^57^ Trastuzumab is a monoclonal antibody targeting HER2 and is used in patients with breast cancers overexpressing this receptor (Table 2).^58, 59^ Trastuzumab inhibits the activity of HER2, which forms heterodimers with other tyrosine kinase receptors (i.e., EGFR, HER3 and HER4) and promotes tumorigenesis.^60^ From a clinical trial of 469 women with metastatic breast cancer overexpressing HER2, combinational treatment with trastuzumab and standard chemotherapy attenuated disease progression compared to standard chemoptherapy alone (i.e., median, 4.6 vs. 7.4 months).^61^ The objective response rate (i.e., 32 vs. 50%) and survival time (i.e., median, 20.3 vs. 25.1 months) were also improved by the addition of trastuzumab to the chemotherapy.^61^ A combination of pertuzumab, trastuzumab, and chemotherapy (i.e., docetaxel) improved the median overall survival time (i.e., 40.8 vs. 56.5 months) compared to trastuzumab-only plus chemotherapy.^62^ A conjugate drug of a HER2 monoclonal antibody and a cytotoxic drug, ado-trastuzumab emtansine, prolonged progression-free survival and overall survival with lower adverse effects compared with a combination of lapatinib and chemotherapy (Table 2).^63^

**Table 2:** Targeted therapies in breast cancer

Target gene	Alteration	Drug type	Examples
*HER2/ERBB2*	Amplification/mutation	HER2 inhibitor	Trastuzumab, ado-trastuzumab emtansine, pertuzumab, lapatinib
*ER*	−	ER inhibitor	Tamoxifen
	Amplification	ER downregulator	Fulvestrant
*EGFR*	Amplification/mutation	EGFR inhibitor	Gefitinib, erlotinib, afatinib, osimertinib, olmutinib, cetuximab
*PI3-K*	Amplification/mutation	mTOR inhibitor	Rapamycin, everolimus
*AKT1/2/3*	Amplification		
*PTEN*	Mutation/deletion		
*mTOR*	Amplification		
*KRAS*	Amplification/mutation	BRAF, MEK inhibitor	Vemurafenib, trametinib
*BRAF*	Amplification/mutation		
*NF1*	Mutation		
*CDKN1B*	Alteration	CDK4 inhibitor	Palbociclib
*CCND1*	Amplification		
*BRCA1/2*	Mutation/deletion	PARP inhibitor	Olaparib
*ATM*	Mutation		
*ATR*	Mutation		

#### Challenges of targeted breast cancer therapies

The identification of driver genes in breast cancer increases the likelihood of matching the correct, most effective drug to the right patient. Only a few genes, however, have been validated to act as driver genes. *BRCA1/2, estrogen receptor alpha (ESR1), HER2, PI3-K/Akt/mTOR, Egfr, cyclin dependent kinase 4 (CDK4)/Rb, Ras/Raf/mitogen-activated protein kinase (MEK)* are known to be critical in breast cancer therapy (Table 2).^64, 65^ For instance, somatic mutations of *PI3-K* occur in more than 10% of all breast cancers^65^ and *Akt1* and *Akt3* mutations and *PTEN* deletion contribute to the activation of the PI3-K pathway.^28, 66^ Nevertheless, approximately 50% of the familial relative risk (the ratio of the risk of disease for a relative of an affected individual to that for the general population) of breast cancer is still unexplained.^17^ Couch et al. estimated that contributions of genes including *BRCA1/2, TP53, PTEN, LKB1, CDH1*, and known/predicted single-nucleotide polymorphisms in breast cancer, and the current knowledge of genetic variations, only covered half of the breast cancer risk.^17^

Variants of uncertain significance (VUS) add another layer of complication in breast cancer treatment. VUS refers to changes in a normal gene sequence for which the clinical association with disease is unclear.^67^ Although many efforts have been made to evaluate and classify genetic variants, including missense, intronic, and small in-frame insertions and deletions,^68–70^ the rarity of the individual VUS makes interpretation difficult because of insufficient statistical power.

Furthermore, some breast cancer patients lack good target proteins for therapy. Triple-negative breast cancer (TNBC), for example, is negative for ER and progesterone receptor (PgR) and lacks HER2 amplification and therefore cannot be treated with classic endocrine therapy or HER2-targeted therapy.^71, 72^ The loss of HER2 expression in metastatic tumors compared with HER2-amplified primary breast cancers is frequently observed, and ER-positive/PgR-positive/HER2-amplified tumors become TNBC after chemotherapy.^55, 73, 74^ Although alternative molecular targets, such as EGFR, which is frequently amplified and is related to poor prognosis,^75, 76^ are being elucidated (Table 2), TNBC has only a small number of therapeutic options, and remains a difficult type of cancer in the field of breast cancer therapy.^77^

#### Lung cancer and targeted therapies

FDA approval of gefitinib, an epidermal growth factor receptor (EGFR) inhibitor,^78^ accelerated target-based therapy in lung cancer patients replacing cytotoxic chemotherapy for first-line therapy.^79, 80^ For patients with EGFR-activating mutations (exon19del or L858R), erlotinib^81, 82^ also performed better than conventional chemotherapies, such as cisplatin. EGFR-targeted therapy was combined with cytotoxic drugs as a combination therapy that showed improved progression-free survival.^83, 84^ However, almost all patients treated with the EGFR inhibitors acquired resistance to the drugs due to secondary EGFR mutations such as T790M.^85, 86^ Second-generation EGFR inhibitors (e.g., afatinib^87, 88^) were designed to target mutant EGFR better than the wild-type receptor. Third-generation EGFR inhibitors, including osimertinib and olmutinib, irreversibly bind to EGFR T790M and have been approved for use in the U.S. and South Korea, respectively (Table 3).^89–91^

**Table 3:** Targeted therapies in lung cancer

Target gene	Alteration	Drug type	Candidate
*EGFR*	Amplification	EGFR inhibitor	Gefitinib, erlotinib, afatinib, osimertinib, olmutinib
*ALK*	Translocation/mutation	ALK inhibitor	Crizotinib, alectinib, ceritinib
*MET*	Amplification	MET/ROS1 inhibitor	Crizotinib, cabozantinib
*RET*	Amplification/mutation		
*ROS1*	Fusion		
*PD-L1*	−	PD-1 inhibitor	Nivolumab, pembrolizumab
*HER2*	Amplification	HER2 inhibitor	Trastuzumab, afatinib, dacomitinib
*BRAF*	Amplification/mutation	BRAF inhibitor	Vemurafenib, dabrafenib

Crizotinib is an FDA-approved inhibitor of anaplastic lymphoma kinase (ALK), Ros proto-oncogene 1 (ROS1), and Met proto-oncogene (MET; Table 3). ALK is a cell surface protein that stimulates signaling pathways, such as the Ras/Raf/MEK, PI3-K/mTOR, and Janus kinase (JAK)/signal transducer and activator of transcription (STAT) pathways,^92^ and is activated by gene translocation and fusion with other genes.^93–98^ ROS1 is an orphan receptor tyrosine kinse (RTK) activated by chromosomal rearrangement and fusion with other genes.^99^ MET, another type of RTK, is overexpressed/amplified or exhibits an exon 14 skip-mutation in non-small-cell lung cancer (NSCLC) patients.^100^ Crizotinib has shown its superiority over standard chemotherapy in ALK-positive lung cancer patients^101, 102^ and ROS1-rearranged NSCLC patients.^103^ Lung adenocarcinoma patients harboring the MET exon 14 splice site mutation also responded to crizotinib.^104^ However, ALK mutations, such as R1174L, L1196M, and R1275Q, conferred resistance to crizotinib and led to the development of second-generation ALK inhibitors. FDA has approved the use of ceritinib,^105^ which targets the L1196M gatekeeper mutation, and alectinib,^106^ which targets the R1174L, L1196M, and R1275Q mutations.

BRAF is a signaling protein activated by various RTKs. In NSCLC, 2–4% of patients possess BRAF V600 mutations.^100^ Although BRAF inhibitors (vemurafenib and dabrafenib) were originally developed for the treatment of melanoma, recent clinical trials with the inhibitors showed potential in BRAF V600 mutant NSCLC patients (Table 3). Vemurafenib resulted in tumor regression in the majority (14 of 19) of NSCLC patients, and the objective response rate was 42%.^107^ In a phase 2 trial, dabrafenib treatment with trametinib, a MEK inhibitor, reached 63% overall response in BRAF V600E-mutant NSCLC patients, who had documented tumor progression after previous platinum-based chemotherapy.^108^

Immunotherapy has received substantial attention recently as a cancer therapy. Unlike other therapies, the goal of immunotherapy is to boost or restore the ability of immune cells to kill tumor cells.^109^ Tumor cells suppress and evade the immune system through interactions between the programmed cell death protein 1 (PD-1) of T-cells and the PD ligand 1 (PD-L1) of tumor cells.^110^ Two monoclonal antibodies against PD-1 (Table 3), including nivolumab^111–114^ and pembrolizumab,^115, 116^ have received FDA approval for second-line treatment against NSCLC that express PD-L1.

#### Challenges of targeted lung cancer therapies

The war against drug resistance is probably the most difficult challenge in lung cancer treatment. Clonal evolution, the accumulation of genetic and epigenetic changes over time in individual cells,^117, 118^ is now believed to be the root of drug resistance.^119, 120^ Biopsies that were taken after the failure of rociletinib targeting EGFR mutant (T790M)-expressing lung cancer^121^ showed that at least a portion of the resistant tumor still expressed the T790-wild-type protein.^120^ The wild-type clones existed before treatment with rociletinib. Piotrowska et al. concluded that combination treatment using rociletinib targeting mutant EGFR T790M clones and other drugs targeting wild-type EGFR T790 are required to further improve the drug response rate and final outcomes.^120^ However, when first-generation EGFR inhibitors (gefitinib and erlotinib) and third-generation EGFR inhibitors (rociletinib/CO-1686, osimertinib/AZD9291, olmutinib/HM61713 and WZ4002) were used in combination, a new mutation of C797S emerged and complicated the therapeutic options.^122^ Studies showed that if the C797S mutation was on a different allele of T790M, combination treatment with gefitinib and WZ4002 inhibited EGFR signaling. In contrast, if C797S and T790M were on the same EGFR allele, the combination of gefitinib and WZ4002 was not effective.^122^ Monitoring changes in cancer cells at the molecular level will be helpful in preventing and resolving drug resistance in lung cancer.

#### Melanoma and targeted therapies

BRAF in the mitogen-activated protein kinase (MAPK) pathway (Ras-Raf-MEK-ERK) is constitutively activated by mutations in 40% of melanomas.^123^ The most common mutations are V600E and V600K, representing 73 and 19%, respectively.^124^ The growth of BRAF V600E-expressing melanoma can be inhibited directly by vemurafenib^125^ or dabrafenib (Table 4).^126^ Drug resistance, also called “bypass tracks”, is increasingly relevant as targeted therapy emerges.^127^ Patients with BRAF mutations acquired resistance due to increased expression and phosphorylation of platelet-derived growth factor receptor beta (PDGFβ) and N-Ras.^128^ The MEK inhibitor, trametinib, also suppresses BRAF V600E-expressing or V600K-expressing melanomas by targeting the BRAF downstream MAPK pathway (Table 4).^129^ Because drug monotherapies commonly result in resistance,^128, 130^ combination treatment with a BRAF inhibitor and a MEK inhibitor has been prescribed to increase the patient’s response rate and also lengthen their survival time.^131^ Especially, combination therapy as a first-line approach increased overall survival rates in a clinical trial.^132^ In patients with BRAF V600 mutations, a combination of cobimetinib (MEK inhibitor) and vemurafenib treatment showed median overall survival of 22.3 months, compared with 17.4 months by placebo and vemurafenib treatment.^132^

**Table 4:** Targeted therapies in melanoma

Target gene	Alteration	Drug type	Candidate
*BRAF*	Amplification/mutation	BRAF inhibitor	Vemurafenib, dabrafenib
*MEK*	Amplification/mutation	MEK inhibitor	Trametinib, cobimetinib
*CTLA-4*	Amplification	CTLA-4 inhibitor	Ipilimumab
*PD-1*	−	PD-1 inhibitor	Nivolumab, pembrolizumab

Melanoma cells express the CTLA-4 and PD-1 immune receptor proteins that are normally expressed in T-cells. Because each of these two proteins can inhibit activation of T-cells and down-regulate the immune response, the abnormal expression of CTLA-4 and PD-1 in tumor cells is suggested as a molecular mechanism of immune evasion in tumors.^133^ Ipilimumab, a CTLA-4 monoclonal antibody, was approved by the FDA for treatment of patients with metastatic melanoma (Table 4).^134, 135^ The PD-1 monoclonal antibodies, nivolumab^136–138^ and pembrolizumab,^139^ were also effective against metastatic melanoma (Table 4). Ipilimumab and nivolumab are also used in combination to treat melanoma.^140^

#### Challenges of targeted melanoma therapies

Similar to other types of cancer, one of the greatest challenges in melanoma treatment is the relapse and development of resistant disease after therapy. Recently, even patients who have undergone immunotherapy were shown to acquire resistance to PD-1 blockade in melanoma.^141^ Zaretsky et al. reported a delayed relapse of patients who had had initial tumor regression induced by continuous pembrolizumab treatment. The analyses of biopsies showed that JAK1/2 truncating mutations resulted in loss of PD-L1 expression and changed the molecular profile of the melanoma.^141, 142^ Overall, although immunotherapy has become a promising and unique strategy for cancer treatment, an integrative strategy should be prepared to prevent drug resistance.

## Tools for precision oncology

The demand for new diagnostic and treatment tools has been driven by precision oncology. In particular, the use of liquid biopsies and patient-derived xenograft (PDX) models has received considerable attention from researchers and clinicians. For precision diagnosis, having new diagnostic platforms like liquid biopsies is crucial because this type of assay can gather information from patients in a manner that is minimally invasive. For precision therapy, testing drugs in a paradigm like the PDX model is beneficial because this model can be used to represent tumors of patients before drugs are prescribed.

### Liquid biopsies

A biopsy is an examination of tissue obtained from a living body to discover the presence, cause, or extent of a disease. Although biopsies have become more important as the field of precision oncology continues to expand, sampling some types of tumors is still difficult and can result in diagnostic errors. To address this problem, biofluid samples, including serum, plasma, saliva, urine, and cerebrospinal fluid, are now being used to screen for tumors, characterize molecular features, and analyze tumor types.^143–146^ Liquid biopsies can provide clear information regarding the genetic makeup of each tumor. Because liquid biopsies are relatively non-invasive, clinicians can repeat sampling and monitor disease progression over time without performing solid-tissue biopsies. Circulating tumor cells (CTCs) and cell-free DNA (cfDNA) are promising components of liquid biopsies.

CTCs are cancer cells that are shed into the vascular system from the primary tumor and are circulating around the body in the blood.^147^ CTCs have been detected in patients with metastatic tumors at an average concentration of 1–10 cells/ml, but are extremely rare in individuals without tumors or with non-malignant tumors.^143, 148–150^ Circulating non-tumor epithelial cells in the blood of patients undergoing surgery and the difficulty in identifying markers of CTCs pose challenges to this technology. However, because dynamic changes occur in surface markers of CTCs, analysis of DNA/RNA from CTCs can enable clinicians to predict tumor progression and drug susceptibility of the patient.^146, 151–154^ A clinical trial with CTCs showed its promise as a prognostic marker and limitation as an indicator of changing chemotherapy.^155^ The trial divided patients with metastatic breast cancer into four groups. Patients whose CTC number was not increased at baseline remained on initial therapy (arm A), and patients whose CTC numbers had been increased, but later decreased after 21 days of therapy, also remained on the initial therapy (arm B). Patients whose CTC numbers were consistently increased were randomly assigned to maintain initial therapy (arm C1) or changed to an alternative therapy (arm C2). Overall survival rates between arms A, B, and C (sum of C1 and C2) showed significant differences between groups (i.e., 35 vs. 23 vs. 13 months, respectively). However, no difference was observed between the overall survival rates of arms C1 and C2. This result indicates that CTC is a strong prognostic marker of overall survival in patients with metastatic breast cancer, although changing chemotherapy options based on CTC does not prolong the overall survival rate.^155^ In summary, monitoring the efficacy of adjuvant therapies is feasible with CTC-based liquid biopsies, analyses of CTCs will be one of the key players in precision diagnosis.

cfDNA refers to tumor DNA released from primary cancers into the biofluids of cancer patients.^146^ The majority of cfDNA is derived from cells that have undergone apoptosis or necrosis and then release fragments of DNA of approximately 150–180 bp in length.^144, 146^ Similar to acquiring CTC, tumor cell-derived cfDNA is difficult to obtain because of the extensive amount of cfDNA that is also released from non-malignant cells.^156^ Nevertheless, cfDNA could provide a better diagnostic tool than CTCs from the same patient to detect mutations.^157^ Because cfDNA could be used to monitor clonal evolution and emergence of drug resistance,^158^ this type of analysis might assist clinicians in making tailored therapeutic decisions for cancer patients in the future.

### PDX models

The PDX model uses actual patient tumor fragments that have been sectioned from the cancer patient and implanted into immunodeficient mice.^159^ By treating mice harboring the PDX tumor fragment, the efficacy of a drug can be predicted before being prescribed to the actual patient. Thus PDX is a platform that provides evidence-based guidelines in choosing the correct and most effective drug to prescribe to a patient. For preclinical drug development, the PDX model overcomes the important limitation of using conventional cancer cell lines, which have developed characteristics that do not accurately reflect the actual cancer patient tumor. Conventional cell line-based xenografts lack the broad diversity and heterogeneity of cancer.^160^ In contrast, the PDX model preserves the heterogeneity and microenvironment of the original tumor after being passaged in mice.^159, 160^ In 1985 the PDX model was confirmed to have good predictive value in showing that drug responses from PDX models corresponded very well with the response from patients.^161^ The tumor heterogeneity of the PDX was shown to be well preserved in patient-derived tumor cells (PDTC).^162^ These results support the potential of the PDTC-PDX pipeline for drug development. Although the lack of functional immune reactions in this model is a limitation, humanized mice that mimic the human immune system and resultant microenvironment allow researchers to better understand translational oncology.^163^ Overall, this clinically relevant mouse model should be beneficial in drug development and precision therapy for cancer patients.

Establishing PDX models worldwide reflects the high expectations of translating preclinical research to the clinic. Novartis, a large pharmaceutical company (Switzerland), has established about 1000 PDX models expressing a diverse pool of driver mutations in cancer.^164^ EurOPDX, a European consortium for PDX, has established more than 1500 subcutaneous and orthotopic PDX models^165^ and the Jackson Laboratory (ME, USA) has created about 550 PDX models.^165^ The US–China (Henan) Hormel Cancer Institute (Zhengzhou, China) has established unique PDX models that include Wilms’ tumor and esophageal cancer models. Dana-Farber Cancer Institute (MA, USA) established a Public Repository of Xenografts (PRoXe), which includes PDXs of leukemia and lymphoma.^166^ Of particular note, EurOPDX launched cBioPortal, where information on models and their molecular annotation have been opened to the public to provide a platform of PDX studies.^167^

## Limitations and prospects of precision oncology

Despite the growing enthusiasm and enormous investment in precision oncology, empirical evidence and verification is still critically needed showing that precision therapy is significantly better than conventional treatments.^168, 169^ Results from one of the first clinical trials based on precision oncology were not promising.^170^ In this trial, the genomic information of patients was analyzed and patients who had targetable driver mutations were subjected to precision therapy. Unfortunately, the use of molecular-targeted drugs did not result in improved progression-free survival compared to treatments based on the clinicians’ choice (i.e., randomized trial). Another study that enrolled patients with different types of tumors, including colon, thyroid and ovarian cancer expressing the BRAF V600 mutation, showed similar results.^107^ In this study, vemurafenib, an FDA-approved drug against melanoma, was only effective in some of the non-melanoma patients. These results suggest that prescription of drugs against a certain type of cancer does not guarantee success in treatment of other types of cancers although they harbor the same mutation/alteration on the target protein.

Several factors could have led to the lack of success of the current precision oncology-based trials.^171^ One factor could be the lack of specific molecular-targeted drugs. Drugs are not yet available for many drivers in carcinogenesis at least partly due to the tremendous cost in money and time. Furthermore, many drugs are so toxic that clinicians are forced to reduce the dose, which results in only a partial inhibition of the targeted pathway giving the tumor the opportunity to develop resistance to the drugs. Other factors include tumor heterogeneity and constant evolution.^119, 172–174^ In addition, the genomic signature from one part of the tumor measured at a certain time point likely does not represent other parts or different time points of tumor development. These features pose a huge hurdle to precision oncology.

To address tumor heterogeneity and evolution, the National Cancer Institute recently revealed a new trial design of genomically informed precision therapy, referred to as the NCI-MATCH (Molecular Analysis for Therapy Choice) Trial.^175, 176^ NCI-MATCH aims to identify ‘actionable mutations’ and test whether a drug or drug combinations are active against specific molecular abnormalities.^176^ The multi-arm phase 2 trial initially aimed to screen 3000 patients and enroll 1000 adults with advanced solid tumors for which standard therapy has not yet been developed. The initial trial with 10 treatment arms has completed accrual and patient recruitment was closed in November 2015 for planned interim analysis. Based on the low number of actionable mutations and the enrollment number exceeding expectations from the interim analysis, the trial reopened on May 31, 2016, extending treatment arms to 24 and aiming to screen 6000 patients.^177^ Genetic variants of the patients will be analyzed for 143 genes and the patients assigned to one of several different treatment groups.^175^ The hope is that this new design will increase the flexibility of clinical decisions and improve the overall outcome of the patients.

One of the most promising and successful examples of precision oncology is the treatment of chronic myeloid leukemia (CML) with imatinib.^10^ The high proportion of the clonal BCR–ABL translocation in CML enabled almost all CML patients to benefit from imatinib.^178^ Likewise, finding concurrent driver mutations or clonal markers will benefit a large group of people. The hope is that predicting/monitoring changes in cells and dividing patients with subgroups before or at the point of cancer relapse will improve clinical outcomes.

Pan-cancer precision diagnosis and therapy are emerging. For instance, pembrolizumab, a PD-1 inhibitor, is approved by the FDA for treatment of metastatic melanoma,^179^ metastatic NSCLC,^180^ Hodgkin’s lymphoma,^181^ and metastatic head and neck squamous cell carcinomas.^182^ Although the proportion of patients who are eligible for the treatment is lower than that of imatinib-CML cases, this target-based approach provided a better treatment option compared to adjuvant chemotherapy. Furthermore, RTK inhibitors also aim to cover patients across various cancer types. One of the newest approaches is a fibroblast growth factor receptor (FGFR) inhibitor that directly targets its genetic alterations. BGJ398, a FGFR1-3 inhibitor, was tested in patients with advanced solid tumors including lung, breast, bladder, colon, and liver.^183^ The phase 1 trial showed that BGJ398 exhibits anti-tumor activity against FGFR1-amplified NSCLC and FGFR3-mutant bladder cancer.^183^ For precision diagnosis, eligible patients for targeted therapies will be screened and for precision therapy, the targeted drug(s) will be administered at a correct dose to benefit each individual.

The advances in lipidomics, proteomics, and metabolomics will aid in the implementation of precision oncology. In the field of lipidomics, arachidonic acid is a signaling precursor that has attracted interest for anti-cancer therapy.^184, 185^ A recent study of lipidomic profiling showed that lung tumors possess higher levels of arachidonic acid-containing phospholipids and phosphatidylinositols compared to normal tissue.^185^ Myc inactivation caused a significant decrease in arachidonic acid and its lipid metabolites.^185^ These results suggest that arachinonic acid and its metabolites can serve as biomarkers in precision diagnosis. In the field of proteomics, cryo-electron microscopy (cryo-EM) has received considerable attention as a tool for “visual proteomics”.^10, 186^ For example, the structure of the ATP-binding cassette subfamily G2 (ABCG2), a human multidrug transporter, revealed that two cholesterol molecules are bound to a hydrophobic pocket between the transmembrane domains.^187^ The cryo-EM structure provides structural insights of cholesterol recognition and pharmacokinetics of ABCG2 in precision therapy. In the field of metabolomics, gut microbiota possess a large repertoire of metabolizing xenobiotics, small molecules that are foreign to the human body.^188^ For example, irinotecan becomes SN-38, an active topoisomerase inhibitor in the body.^189^ SN-38 is glucuronidated by host liver enzymes and loses its activity (SN-38G). However, bacterial β-glucuronidase hydrolyzes and reactivates SN-38G in the large intestine, causing intestinal damage and diarrhea.^189^ Accumulative knowledge of microbiota in each patient could enhance the efficacy of precision therapy and lower adverse side effects.

To summarize, precision oncology has emerged to improve efficacy, minimize side effects of drugs and avoid drug resistance in cancer therapy. Precision oncology is categorized into two segments, precision diagnosis and precision therapy. In precision diagnosis, mutations of important genes such as *BRCA1/2* have led clinicians to tailor treatment options to individuals. Improvement on genetic tests facilitated molecular target-based precision therapy. In breast cancer, lung cancer, and melanoma, drugs that inhibit important target proteins prolonged progression-free survival and overall survival of the patients compared to genotoxic chemotherapy. Although difficulties due to clonal evolution and cancer heterogeneity still exist, state-of-the-art tools such as liquid biopsies and PDX models will enhance the efficacy of precision diagnosis and precision therapy, respectively. Supported by government-driven projects worldwide, precision oncology will be the standard pipeline of cancer therapy.
